# Molecular Bases of Myopathies and Their Impact on Clinical Practice: Advances and Future Perspectives

**DOI:** 10.3390/ijms27031392

**Published:** 2026-01-30

**Authors:** Martín Campuzano-Donoso, Claudia Reytor-González, Melannie Toral-Noristz, Yamilia González, Daniel Simancas-Racines

**Affiliations:** 1 Facultad de Ciencias de la Salud y Bienestar Humano, Universidad Tecnológica Indoamérica, Ambato 180150, Ecuador; 2 Escuela de Medicina, Universidad Espíritu Santo, Samborondón 0901952, Ecuador; 3 Independent Researcher, Santo Domingo 230101, Ecuador

**Keywords:** myopathies, molecular genetics, next-generation sequencing, precision medicine, therapeutic strategies

## Abstract

Myopathies represent a highly heterogeneous group of primary muscle disorders, traditionally classified based on clinical presentation and histopathological findings. Recent breakthroughs in molecular genetics, immunology, and pathophysiology have revolutionized the understanding, diagnosis, and management of these conditions. Both inherited and acquired forms of myopathy, including structural, metabolic, inflammatory, endocrine, and mitochondrial subtypes, are now recognized to arise from diverse pathogenic mechanisms such as impaired calcium handling, mitochondrial dysfunction, chronic inflammation, altered metabolism, and defective muscle regeneration. The advent of next-generation sequencing technologies has enabled more precise diagnosis of genetic forms, while the discovery of novel molecular biomarkers and immunological signatures offers promising avenues for disease monitoring and stratification across the broader spectrum. Importantly, molecular and mechanistic insights have redefined clinical classifications, allowing for better prognostic predictions and patient-tailored therapeutic approaches. Innovative treatments, including gene therapy, antisense oligonucleotide therapies, immune-modulating agents, metabolic support strategies, and targeted pharmacological interventions, are progressively translating molecular knowledge into clinical applications. However, technical limitations, biological variability, and ethical considerations continue to pose significant challenges to the implementation of precision medicine in myopathies. In this narrative review, we comprehensively discuss the latest molecular findings, their integration into clinical practice, and the emerging therapeutic strategies based on these discoveries. We also highlight current limitations and propose future research directions aimed at bridging the gap between molecular insights and effective, equitable patient care.

## 1. Introduction

Myopathies comprise a broad and heterogeneous group of primary muscle disorders with diverse clinical and genetic profiles, often leading to progressive muscle weakness, wasting, and, in some cases, pain or stiffness [1,2]. These conditions arise from structural or functional abnormalities of muscle fibers, caused by a variety of factors including genetic mutations, inflammatory processes, metabolic defects, or endocrine dysregulation [1].

Historically, the recognition of myopathies evolved significantly during the 19th and early 20th centuries, with seminal contributions from Duchenne, Erb, and Landouzy in describing muscular dystrophies. This era also marked the first clinical descriptions of congenital myopathies (CMs), though their clear separation from other muscle diseases only became possible with the introduction of modern pathological techniques in the mid-20th century [3].

Epidemiologically, inflammatory and endocrine myopathies represent some of the more common types, typically occurring in middle-aged women more frequently than men. Incidence rates of inflammatory myopathies range from 1.16 to 19 per million per year, with prevalence estimates between 2.4 and 33.8 per 100,000 individuals [4]. The incidence of idiopathic inflammatory myopathies (IIMs) is estimated at 0.2–2 per 100,000 person-years, with prevalence ranging from 2 to 25 per 100,000 people; although age and gender patterns are well recognized, ethnic differences remain poorly understood [5]. Among inherited myopathies, dystrophinopathies are the most common, affecting males across all races and ethnicities. Duchenne and Becker muscular dystrophies are the most prevalent subtypes, while mitochondrial myopathies occur in approximately 1 in 4300 individuals, and other hereditary forms are rare [3]. Limb–girdle muscular dystrophies (LGMDs), though more heterogeneous, are also an important group, with an estimated prevalence of 1 in 14,500 to 1 in 123,000, averaging approximately 1 in 50,000 [6]. LGMD encompasses numerous genetic subtypes, many of which remain underdiagnosed due to clinical overlap and variable disease expression. Their frequency underscores the need for greater attention in both research and clinical practice [7].

Genetic mutations impacting muscle integrity can lead to severe consequences such as impaired mobility, respiratory failure, and, in the most severe forms, early mortality. Based on molecular cause and histopathological appearance, these disorders are classified as myopathies or muscular dystrophies [8]. Their clinical heterogeneity often complicates diagnosis, which may be further obscured by extra-muscular manifestations that appear unrelated [9]. Despite their heterogeneity, myopathies share several pathogenic features, including impaired calcium handling, mitochondrial dysfunction, chronic inflammation, and defective muscle regeneration [10].

Traditionally, diagnosis relied on clinical evaluation and histopathological examination of muscle biopsies. While inflammatory myopathies share features such as mononuclear cell infiltrates and muscle fiber necrosis, distinct patterns can help differentiate subtypes [11]. The advent of next-generation sequencing (NGS) has shifted the diagnostic paradigm, particularly in genetic and metabolic myopathies, from a symptom-driven approach toward elucidating the molecular basis of disease. This transition has enhanced diagnostic precision and facilitated the development of molecularly targeted therapies [12].

*This review will examine recent advances in the molecular understanding of myopathies, their integration into clinical practice, and emerging targeted treatments. It will also discuss current challenges, ethical considerations, and future perspectives, emphasizing the translation of molecular discoveries into effective and equitable patient care.*

## 2. Molecular Pathogenesis of Myopathies

### 2.1. Genetic Alterations and Their Functional Consequences

#### 2.1.1. Dystrophinopathies (Duchenne Muscular Dystrophy—DMD and Becker Muscular Dystrophy—BMD)

Duchenne muscular dystrophy (DMD) is one of the most prevalent and severe forms of muscular dystrophy [13], caused by mutations in the *DMD* gene, most often deletions spanning one or more exons [14]. This gene, among the largest in the human genome, which spans over 2 megabases, contains 79 exons, and is located at Xp21 [15,16]. It encodes dystrophin, a critical component of the dystrophin–glycoprotein complex (DGC), which stabilizes the sarcolemma, the plasma membrane of striated muscle cells, by linking the actin cytoskeleton to the extracellular matrix [13,17]. Beyond its structural function, dystrophin anchors neuronal nitric oxide synthase (nNOS) to the membrane, facilitating proper vasodilation and blood flow during muscle activity [18].

Loss-of-function mutations in *DMD* or related genes disrupt the DGC, rendering the sarcolemma fragile and prone to mechanical injury during contraction [13]. The most common pathogenic mechanism is exon deletion, accounting for 60–70% of DMD and 80–85% of Becker muscular dystrophy (BMD) cases [19]. Frameshifting or nonsense mutations (often out-of-frame deletions/duplications) abolish or severely reduce production of the muscle isoform Dp427m, leading to DMD, whereas in-frame mutations typically produce an internally truncated but partially functional dystrophin, causing the milder BMD phenotype [20,21]. In BMD, the degree of residual protein functionality, particularly the preservation of critical domains that maintain linkage between actin and β-dystroglycan, plays a central role in determining disease severity [13,22]. Even when truncated, dystrophin molecules that retain essential binding sites can stabilize the sarcolemma, delaying onset and slowing progression, whereas loss of these domains results in more severe manifestations [23].

The absence of functional dystrophin in DMD weakens the muscle fiber–ECM connection, causing sarcolemmal tears, leakage of muscle enzymes such as creatine kinase into the blood [24,25], and uncontrolled calcium influx that activates cell death pathways [24]. Failure to anchor nNOS exacerbates the pathology by causing functional ischemia [18]. Moreover, loss of dystrophin disrupts redox homeostasis: mislocalization of nNOS and hyperactivation of NOX2 promote excess production of reactive oxygen species (ROS) and reactive nitrogen species (RNS), driving oxidative damage [26]. This sets off a vicious cycle of membrane damage, inflammation, and regeneration failure, ending in progressive muscle wasting [17].

Clinically, dystrophinopathies present with progressive skeletal, respiratory, and cardiac muscle weakness, leading to loss of ambulation, respiratory insufficiency, and cardiomyopathy [27,28]. Cardiac involvement, though often later in onset, is a major cause of morbidity and may be modulated by tissue-specific dystrophin expression patterns, DGC composition in the myocardium, and interactions with cardioprotective proteins [29]. The multifactorial cascade of secondary molecular disturbances underscores the need for therapeutic strategies that address not only the primary genetic defect but also downstream pathophysiological mechanisms [23].

#### 2.1.2. Limb–Girdle Muscular Dystrophies

The LGMDs are a genetically heterogeneous group of autosomally inherited disorders with onset ranging from childhood to adulthood, typically presenting with progressive weakness of the hip and shoulder girdle muscles [30]. Unlike Duchenne or Becker muscular dystrophies, which are X-linked disorders caused by mutations in the *DMD* gene, each LGMD subtype arises from mutations in distinct genes encoding specific muscle proteins [31]. Pathogenic variants have been identified in genes coding for sarcoglycans, dysferlin, calpain-3, and other proteins essential to muscle structure and function [32].

LGMD R1 (also known as calpainopathy) is among the most common forms, caused by autosomal recessive mutations in the *CAPN3* gene encoding calpain-3 [33]. Calpain-3 is a calcium-dependent intracellular protease that, while dispensable for prenatal muscle development, is indispensable for lifelong muscle maintenance [34]. It functions as a sensor of sarcomeric integrity, contributing to repair by regulating protein turnover and removing damaged sarcomeric components [34]. Calpain-3 interacts with titin, a large elastic scaffold protein, which stabilizes the protease and restricts its activity to injury sites, preventing uncontrolled proteolysis while enabling localized sarcomere repair [34,35,36]. In addition, calpain-3 influences transcription factors controlling survival pathways and apoptosis, and participates in sarcomere remodeling by mediating the degradation and disassembly of cytoskeletal or myofibrillar proteins [37].

LGMD R2 results from mutations in the *DYSF* gene, which impair production of dysferlin, a protein essential for maintaining sarcomere stability and enabling efficient membrane repair [38]. Clinically, LGMD R2 often presents with distal muscle weakness but progressively affects proximal muscles as well [39]. Dysferlin’s role in rapid membrane resealing after injury highlights a disease mechanism centered on defective repair rather than primary structural fragility [40].

The contrasting examples of calpain-3 and dysferlin show the diverse functions of proteins implicated in LGMD pathogenesis. Calpain-3 acts as a “sensor” of sarcomeric integrity, while dysferlin mediates membrane repair, together emphasizing that muscle health depends on active, ongoing maintenance processes rather than static structural stability alone. Disruption of these processes leads to progressive muscle damage. This broader understanding shifts therapeutic strategies from solely replacing missing proteins to enhancing intrinsic repair mechanisms, modulating proteolytic pathways, or restoring dynamic muscle homeostasis [41,42,43].

#### 2.1.3. Congenital Myopathies

CMs comprise a clinically, genetically, and histologically diverse group of disorders that primarily affect skeletal muscle. They are defined by characteristic histopathological features on muscle biopsy, which distinguish them from other neuromuscular conditions, and are caused by inherited defects in structural or functional muscle proteins [44]. Classification is traditionally based on biopsy findings, with over 40 subtypes identified and more than 30 causative genes reported [45]. These genes frequently encode proteins involved in excitation–contraction coupling (ECC), calcium handling, or sarcomeric architecture [46].

Mutations in ryanodine receptor 1 (RYR1), encoding the ryanodine receptor 1 calcium-release channel, represent the most common genetic cause of CMs [47]. RyR1 is a key component of skeletal muscle ECC, linking the terminal cisternae of the sarcoplasmic reticulum (SR) to T-tubules and mediating rapid calcium release in response to action potentials [46]. Pathogenic RYR1 variants disrupt this process, leading to reduced or dysregulated calcium release and impaired contraction [48]. These mutations can decrease protein stability, alter channel gating, or produce “leaky” channels, causing calcium imbalance [49]. Clinically, RYR1 mutations are associated with central core disease (CCD) and multiminicore disease (MmD). In CCD, hallmark findings include mitochondrial depletion, reduced oxidative enzyme activity, and well-defined central “cores” running along myofibrils [50]. In contrast, MmD is characterized by multiple small, poorly defined areas of sarcomeric disorganization (“minicores”) visible on biopsy, often accompanied by generalized muscle weakness, scoliosis, and respiratory involvement [51]. Importantly, RYR1 mutations predispose to malignant hyperthermia, a potentially fatal hypermetabolic crisis triggered by agents such as volatile anesthetics or caffeine [48].

Another major CM gene, *ACTA1*, encodes α-actin, the principal component of the thin filament in sarcomeres, essential for myosin-mediated contraction [52]. *ACTA1* mutations account for roughly 25% of nemaline myopathy cases and up to 50% of severe early-onset forms, often arising as de novo heterozygous missense variants [52,53]. These mutations impair force generation by disrupting actin–myosin interactions [46].

Recessive mutations in *SELENON* (formerly SEPN1) cause specific forms of MmD [54]. Selenoprotein N, encoded by *SELENON*, is an endoplasmic reticulum glycoprotein involved in calcium homeostasis and protection against oxidative stress (OS) [55,56]. It interacts with *RYR1* and modulates the expression of ECC-related transcripts, including *RYR1* and *ATP2B2* (plasma membrane Ca^2+^-ATPase) [57]. Loss of selenoprotein N alters calcium regulation and OS responses, linking oxidative injury to calcium dysregulation in CM pathogenesis.

A recurring molecular theme in CMs is the disruption of ECC and calcium balance [58]. The interplay between *RYR1* and *SELENON* exemplifies how defects in distinct proteins (one central to calcium release, the other to OS control) can converge on a shared pathogenic pathway. This underscores the multifactorial nature of CM pathogenesis, where diverse genetic lesions ultimately disturb common molecular networks governing muscle contraction and maintenance [56,57,58].

#### 2.1.4. Mitochondrial Myopathies

Mitochondrial myopathies are progressive muscle disorders caused by defects in oxidative phosphorylation (OXPHOS), the primary pathway for ATP generation in mitochondria. Energy deficiency in skeletal muscle, a tissue with high metabolic demand, leads to the hallmark clinical manifestations [59].

These disorders can arise from mutations in either mitochondrial DNA (mtDNA) or nuclear DNA (nDNA) [60]. Mitochondrial function depends on both genomes: the 16.6 kb mtDNA encodes 37 genes (13 polypeptides forming core subunits of respiratory chain complexes I, III, IV, and V, as well as 22 tRNAs and 2 rRNAs) while the nuclear genome encodes the majority of mitochondrial proteins, including OXPHOS subunits, complex assembly factors, and proteins critical for mtDNA maintenance [61,62,63].

Pathogenic mtDNA mutations include point mutations affecting protein-coding or tRNA genes, and large-scale deletions [64]. A distinctive feature of mtDNA genetics is heteroplasmy, the coexistence of mutant and wild-type mtDNA within the same cell [65]. Clinical symptoms typically emerge once the mutant load surpasses a threshold, often around 70% in the most common disorders [66]. This threshold effect, along with the dynamic nature of mtDNA populations during development, explains the variable expressivity and delayed onset frequently observed, even within the same family [65]. Consequently, accurate prognosis requires not only mutation identification but also quantification of the mutant load [59,65].

Mutations in nDNA can cause primary mitochondrial defects or lead to secondary mtDNA abnormalities, such as depletion or multiple deletions [59]. Over 254 nDNA-encoded genes have been implicated in mitochondrial disorders [60]. In mitochondrial myopathies, tissues with high energy demands, including skeletal muscle and brain, are most susceptible to damage [67]. Histopathologically, muscle biopsies may reveal ragged-red fibers (RRFs), representing abnormal mitochondrial accumulation, and cytochrome c oxidase (COX)-negative fibers, both indicative of OXPHOS dysfunction [68].

Understanding the genetic and molecular basis of mitochondrial myopathies is crucial for accurate diagnosis, prognostication, and therapeutic development. These conditions exemplify how complex genetic interactions, between mtDNA and nDNA, heteroplasmy, and threshold effects, translate into diverse phenotypes. The key genetic alterations and pathogenic mechanisms of mitochondrial myopathies, alongside other major myopathy subtypes, are summarized in Table 1.

#### 2.1.5. Other Key Myopathy Subtypes: Metabolic, Endocrine, and Inflammatory Myopathies

In addition to the hereditary structural and mitochondrial conditions previously addressed, metabolic, endocrine, and inflammatory myopathies represent widespread clinical categories that contribute significantly to the landscape of muscle disease. While their origins differ, these subtypes often utilize overlapping molecular pathways during disease progression [69].

Metabolic myopathies are characterized by disruptions in the pathways that govern energy synthesis and the utilization of substrates, particularly glycogen, purines, and fatty acid beta-oxidation [70]. Notable examples include disorders of lipid metabolism, such as carnitine palmitoyltransferase II deficiency, and glycogen storage diseases like McArdle disease [70]. The molecular failure to generate sufficient ATP during exertion results in symptoms like chronic myalgia, exercise intolerance, and rhabdomyolysis [71]. These conditions are driven by impaired metabolic flux, oxidative stress, and secondary mitochondrial failure. Although genetic testing has revolutionized diagnostic accuracy, treatment remains largely supportive, emphasizing dietary management, the avoidance of metabolic triggers, and occasional enzyme or substrate replacement therapy [70,72].

Endocrine myopathies are the result of hormonal imbalances that interfere with muscle fiber composition, protein turnover, and general metabolism [73]. Thyroid dysfunction is the most prevalent cause; hyperthyroidism typically promotes muscle weakness through protein catabolism, whereas hypothyroidism is associated with stiffness and elevated creatine kinase levels [74]. Other triggers include glucocorticoid excess, diabetes mellitus, and adrenal or parathyroid disorders. The underlying molecular pathology involves dysregulated calcium handling, mitochondrial impairment, and altered gene transcription [74,75]. Significantly, these conditions are often reversible through the correction of the primary endocrine disorder [74].

Inflammatory myopathies, such as dermatomyositis, polymyositis, and inclusion body myositis, involve muscle damage mediated by the immune system [76]. The pathogenesis is a result of a complex relationship between the innate and adaptive immune systems, featuring T-cell-induced myofiber destruction, cytokine signaling, and autoantibody-triggered complement activation [69]. Molecularly, these diseases are identified by the upregulation of interferon-stimulated genes, activation of the NF-kB pathway, and the development of fibrosis [76]. Biomarkers like myositis-specific autoantibodies are essential for clinical stratification. Although immunosuppressive therapies are the standard of care, persistent fibrosis and refractory cases continue to present clinical difficulties [77].

Ultimately, these myopathy categories illustrate that disparate etiologies often converge on common molecular disruptions, including calcium dysregulation, impaired energy production, and defective muscle regeneration. Recognizing these shared mechanisms provides a unified framework for understanding myopathy and highlights the potential for diagnostic and therapeutic strategies that target these specific biological pathways.

### 2.2. Common Molecular Mechanisms

Beyond specific genetic alterations, myopathies often converge on a set of common molecular mechanisms that collectively contribute to muscle damage and dysfunction, irrespective of the primary genetic cause [8].

#### 2.2.1. Impaired Calcium Handling

Alterations in SR function are a common pathogenic feature across diverse myopathies [16,46,78]. During ECC, the triad, formed where T-tubules interface with the SR, mediates the rapid release of calcium ions required for muscle contraction. Depolarization of the T-tubule activates dihydropyridine receptors (DHPRs), which mechanically couple to the *RYR1* channel in the SR, triggering calcium release into the cytoplasm [46,79]. Mutations in *RYR1*, the skeletal muscle-specific calcium release channel, can directly disrupt ECC, leading to distinct clinical phenotypes: malignant hyperthermia from excessive channel activation, CCD from leaky channels and SR calcium depletion, and multi-minicore disease from reduced channel expression [80]. In many *RYR1*-related myopathies, gain-of-function mutations cause chronic calcium dysregulation [81].

Calcium imbalance also plays a major secondary role in myopathies not directly caused by *RYR1* mutations. In DMD, loss of dystrophin compromises sarcolemmal integrity, altering action potential-elicited RyR1-mediated calcium release and producing ECC deficits [82,83]. The destabilized membrane also increases permeability to extracellular calcium, causing sustained intracellular calcium elevation that activates degradative pathways and promotes muscle fiber necrosis [83].

In DMD, RyR1 dysfunction is further exacerbated by abnormal S-nitrosylation, which weakens the stabilizing FKBP12–RyR1 interaction, enhancing calcium leak from the SR [84]. The resulting cytosolic calcium overload drives proteolysis, mitochondrial dysfunction, and cell death. Therefore, calcium dysregulation can arise as a primary defect (e.g., pathogenic *RYR1* variants) or a secondary consequence (e.g., dystrophin deficiency in DMD) [83,85]. Given calcium’s central role in contraction, signaling, and cell survival, therapeutic strategies targeting calcium channels or SR regulation hold potential for application across multiple myopathy subtypes.

The clinical presentation of *RYR1*-related myopathies is highly diverse, ranging from mild exertional rhabdomyolysis to severe phenotypes such as fetal akinesia or multi-minicore disease with respiratory insufficiency [86,87]. This variability reflects the nature of the underlying mutations: dominant gain-of-function variants typically increase channel activity, whereas severe loss-of-function mutations reduce protein levels or stability [86,88]. Importantly, studies of congenital myopathies with different genetic origins have revealed converging molecular signatures. For instance, reduced expression of not only *RYR1* but also *ATP2B2* and miR-22 suggests a shared transcriptional program that contributes to muscle weakness across distinct subtypes [89]. This highlights that while primary mutations initiate pathology, downstream transcriptional dysregulation represents a common pathway that shapes disease progression and clinical overlap [89,90].

Beyond genetic and transcriptional changes, post-translational modifications (PTMs) add another layer of dysregulation in calcium handling [91]. RyR1 is particularly sensitive to oxidative stress, and modifications such as S-nitrosylation and carbonylation increase channel open probability, promoting calcium leak from the sarcoplasmic reticulum [92]. These PTMs often affect the same regulatory domains that harbor pathogenic mutations, directly linking oxidative stress to functional channel defects [92]. Similar PTM-driven remodeling is observed in acquired conditions, including aging, where dissociation of calstabin1 from the RyR1 complex correlates with reduced muscle force. Thus, PTMs appear to be a convergent mechanism through which both inherited and non-inherited insults impair calcium homeostasis [93]. A complete picture of calcium dysregulation must also include the sarco/endoplasmic reticulum Ca^2+^-ATPase (SERCA) pump, which governs calcium re-uptake into the SR. SERCA function itself is regulated by PTMs, including phosphorylation by GSK3β and modulation of its inhibitor phospholamban (PLB) [94]. Together, these multilevel regulatory disturbances underscore that calcium imbalance in myopathies is not the result of a single defect but rather the convergence of multiple pathogenic processes.

#### 2.2.2. Mitochondrial Dysfunction and Oxidative Stress

OS arises from an imbalance between pro-oxidant and antioxidant systems, leading to the excessive accumulation of ROS and RNS [95]. While moderate ROS levels have physiological signaling roles, sustained overproduction, coupled with impaired antioxidant defenses, causes oxidative damage to macromolecules and disrupts cellular homeostasis. Also, excessive depletion of oxidants can induce reductive stress, impairing mitochondrial function, cell proliferation, and contributing to pathologies such as cardiomyopathy [96].

Tissues with high metabolic demands, such as skeletal muscle, heart, and brain, are particularly vulnerable to OS, explaining why mitochondrial disorders often present with myopathy [97]. Mitochondria are the primary source of ROS in most cells, generated as by-products of electron leakage during oxidative phosphorylation [95,97]. These ROS damage mtDNA, proteins, and lipids, thereby impairing mitochondrial function. This dysfunction further amplifies ROS production, creating a self-perpetuating cycle that drives progressive cellular injury and energy depletion [95,98].

OS also acts as a secondary pathogenic mechanism in non-mitochondrial myopathies, notably DMD and BMD, both caused by dystrophin deficiency [15]. In skeletal muscle, nNOS is normally anchored to the sarcolemma via the dystrophin complex [99]. In the absence of dystrophin, nNOS is mislocalized to the cytosol, where its aberrant activity promotes toxic free radical production [100]. Additionally, mechanical stress in dystrophin-deficient muscle activates NADPH NOX2, generating further ROS, while delocalized nNOS increases RNS levels [25]. This redox imbalance damages muscle fibers, accelerates degeneration, and contributes to the loss of mesenchymal progenitor cell functionality through cumulative oxidative injury [101].

The dual role of OS, as both a primary driver in mitochondrial myopathies and a secondary amplifier in structural muscle diseases, highlights its importance as a therapeutic target. Effective interventions will likely need to combine mitochondrial support with antioxidant strategies to break the cycle of ROS-induced damage, potentially slowing disease progression [95,98].

Moreover, mitochondrial myopathies arise from defects in the machinery that maintains mitochondrial function and energy production [59]. Pathogenic variants may occur in either mtDNA or nDNA, leading to diverse clinical syndromes often involving multiple organ systems [59]. For example, mutations in the mtDNA gene *MT-TL1* can cause MELAS syndrome, while nDNA mutations in *POLG* are associated with chronic progressive external ophthalmoplegia (CPEO) [102]. A central regulator of mitochondrial health is peroxisome proliferator-activated receptor-γ coactivator 1α (PGC-1α), which coordinates the expression of nuclear and mitochondrial genes by activating transcription factors such as NRF1/NRF2 and TFAM [103]. PGC-1α also regulates antioxidant gene expression, linking mitochondrial biogenesis with redox balance [104]. Impaired PGC-1α activity has been implicated in myopathies and systemic disorders such as heart failure, highlighting its potential as a therapeutic target [105].

In addition to genetic and transcriptional mechanisms, OS drives PTMs that directly impair muscle protein function [91]. In critical illness myopathy, for instance, oxidative, ubiquitin, and acetyl modifications of myosin alter its structure and compromise contractility, providing a direct mechanistic link between metabolic stress and weakness [106]. OS also interacts with inflammatory pathways to form a self-reinforcing feedback loop: inflammation reduces PGC-1α expression, diminishing antioxidant defenses, while increased OS activates NF-κB, further amplifying inflammation [97,98]. This interplay establishes OS as a molecular bridge connecting genetic, metabolic, and immune pathways, and underscores why therapies that target both mitochondrial function and redox homeostasis may be required to slow disease progression.

#### 2.2.3. Chronic Inflammation and Fibrosis

**Figure 1:**
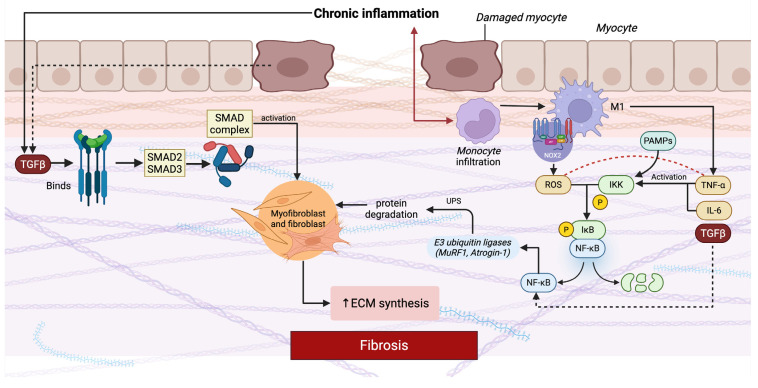
Molecular mechanisms linking chronic inflammation and fibrosis in myopathy. Abbreviations: ECM, extracellular matrix; TGF-β, transforming growth factor beta; SMAD, small mothers against decapentaplegic; NF-κB, nuclear factor kappa-light-chain-enhancer of activated B cells; PAMPs, pathogen-associated molecular patterns; UPS, ubiquitin–proteasome system; TNF-α, tumor necrosis factor alpha; IL-6, interleukin-6; ROS, reactive oxygen species.

Chronic inflammation and fibrosis are central molecular mechanisms driving myopathy progression, largely mediated by persistent activation of transforming growth factor beta (TGF-β) signaling and dysregulation of the nuclear factor-kappa B (NF-κB) pathway (Figure 1) [107,108]. Fibrosis is defined by the excessive deposition of ECM components, which disrupts normal tissue architecture, impairs function, and ultimately replaces contractile muscle fibers with non-functional tissue [109,110].

The schematic highlights two central pro-fibrotic molecular pathways, TGF-β/SMAD and NF-κB, which drive disease progression. In the TGF-β/SMAD pathway, persistent release of TGF-β from damaged muscle fibers and infiltrating immune cells binds to its serine/threonine kinase receptors, triggering phosphorylation of receptor-regulated SMAD proteins (SMAD2/3). These form a complex with SMAD4, translocate to the nucleus, and activate transcription of ECM genes such as collagen and fibronectin, while promoting myofibroblast transdifferentiation. Parallel to this, chronic inflammation activates the NF-κB pathway. Cytokines such as TNF-α and IL-6, released predominantly by infiltrating macrophages and other immune cells, bind to their receptors on myocytes and fibroblasts. Downstream signaling through adaptor proteins and the IKK complex phosphorylates IκB, targeting it for degradation and releasing NF-κB, which translocates into the nucleus. NF-κB activation not only amplifies inflammation by inducing further cytokines and chemokines but also connects directly to muscle atrophy and fibrosis. It stimulates the expression of E3 ubiquitin ligases such as MuRF1 and Atrogin-1, which activate the ubiquitin–proteasome system and promote protein degradation in muscle fibers. In parallel, NF-κB upregulates pro-fibrotic mediators including TGF-β and CTGF, thereby enhancing fibroblast activation and ECM deposition. Excessive ROS further amplify this process by activating NF-κB, promoting cytokine release, and directly stimulating fibroblast activity [107,109,110,111,112,113].

In muscle diseases, chronic inflammation is sustained by immune cell infiltration and aberrant cytokine release, which perpetuate muscle damage while hindering regeneration [107,114,115]. In DMD, for instance, impaired regenerative capacity stems not only from muscle stem cell exhaustion but also from dysfunctional satellite cells. This dysfunction, combined with continuous activation of M2 macrophages, drives persistent ECM accumulation through the release of pro-fibrotic mediators such as TGF-β [113]. However, M2 macrophages also play an important physiological role in muscle repair and remodeling by clearing debris and promoting tissue regeneration. It is the persistence and imbalance of their activity in dystrophic muscle, rather than their presence per se, that shifts their contribution from pro-regenerative to pro-fibrotic [116]. Inflammatory responses that fail to resolve appropriately result in sustained fibrogenic growth factor production, further reinforcing fibrosis [112,117].

TGF-β, a master regulator of fibrogenesis, is upregulated in fibrotic muscle where it promotes myofibroblast transdifferentiation and matrix stabilization [112]. In parallel, NF-κB activation in skeletal muscle fosters protein degradation, amplifies inflammatory signaling, suppresses myofiber regeneration, and contributes directly to fibrotic remodeling [111]. In DMD, NF-κB activity, together with tumor necrosis factor (TNF)-α and IL-6, exacerbates degeneration in a redox-sensitive manner, linking OS to inflammatory pathology [17].

The persistent inflammatory environment in dystrophic muscle, characterized by repeated injury–repair cycles, also supports the coexistence of multiple neutrophil subpopulations, whose maturation, activation, and clearance are tightly regulated to balance antimicrobial and inflammatory functions [107]. However, when this balance is lost, inflammation becomes pathological, creating a detrimental feedback loop where fibrotic tissue progressively replaces functional muscle [17,114].

Fibrosis is increasingly recognized as an actively regulated process rather than a passive accumulation of connective tissue [113]. Central to this regulation are the TGF-β and NF-κB pathways. Canonical TGF-β/SMAD signaling promotes the transcription of pro-fibrotic genes such as collagens and fibronectin, while non-canonical branches (MAPK, PI3K/AKT) further reinforce fibroblast activation and survival [118]. In parallel, NF-κB acts as a hub for both inflammatory and fibrogenic responses by inducing cytokines, proteolytic enzymes, and mediators that suppress muscle regeneration and promote ECM deposition. Together, these pathways orchestrate a transcriptional program that continuously shifts the balance from functional contractile tissue toward fibrosis [113,119].

At the same time, PTMs and protein degradation pathways amplify muscle loss [120]. NF-κB activation shows this link: phosphorylation and ubiquitination of its inhibitor IκB trigger its proteasomal degradation, releasing NF-κB to drive pro-inflammatory and pro-fibrotic transcription [118]. More broadly, chronic inflammation activates the ubiquitin–proteasome system, accelerating the breakdown of structural and contractile proteins. As ECM progressively replaces muscle fibers, the result is a pathological cycle in which inflammatory signaling, transcriptional reprogramming, and proteolytic activity converge to drive sustained atrophy and fibrosis across many myopathies [121].

## 3. Molecular Tools for Diagnosis

### 3.1. Next-Generation Sequencing and Beyond

Advances in genetic research have enabled the timely identification of myopathies and dystrophies, with regional and population-specific mutations now detectable through routine genetic screening. This progress allows for earlier diagnosis, improved management, and targeted counseling for families with a history of these disorders [77,122]. The so-called molecular revolution has transformed diagnostic approaches, moving beyond traditional methods toward genetic and biochemical profiling [123].

NGS has become central to this shift, providing the ability to sequence millions of DNA fragments simultaneously and generating detailed information on genome structure, genetic variation, and gene activity. When combined with clinical evaluation, morphological studies, and imaging modalities such as MRI, NGS significantly enhances diagnostic precision [123]. Both Sanger sequencing and NGS remain valuable in confirming suspected neuromuscular disorders, given the wide range of genetic alterations associated with these conditions [123]. Importantly, neuromuscular diseases are among the most genetically heterogeneous conditions, likely containing the highest proportion of causative Mendelian defects.

NGS technologies encompass different approaches: targeted gene panels (focusing on known myopathy-related genes), whole-exome sequencing (WES) (capturing ~85% of mutations occurring in coding regions), and whole-genome sequencing (WGS) (providing a complete genomic profile, including non-coding elements) [124,125,126]. WGS is particularly useful for atypical or complex phenotypes due to its uniform coverage and ability to identify variants missed by more restricted methods [127]. These strategies have increased diagnostic yield in neuromuscular diseases, facilitated the identification of novel disease-causing genes, and provided crucial insights into rare Mendelian disorders [126].

More recently, long-read sequencing (LRS) has emerged as a complementary technology, addressing limitations of short-read NGS. LRS enables improved phasing of variants, detection of structural rearrangements, copy number variations, short tandem repeat (STR) expansions, and even epigenetic modifications such as DNA methylation [128,129]. Unlike other sequencing platforms, LRS can also capture subtle interruptions or motif variations within STRs, which are clinically relevant since they can modulate disease severity [130,131]. For instance, in myotonic dystrophy type 1 (DM1), LRS can detect CGG interruptions within the expanded CTG repeat in the *DMPK* gene, findings with important prognostic implications [132].

Despite its advantages, both NGS and LRS face limitations. In NGS, challenges include interpreting variants of uncertain significance (VUS), detecting deep intronic or large structural mutations, and distinguishing pathogenic variants from benign polymorphisms [133,134]. Data management and storage add further complexity, given the immense datasets generated. LRS, while powerful, requires specialized DNA extraction protocols, sophisticated bioinformatic pipelines, and remains limited by the relatively small size of available variant databases compared to NGS resources like gnomAD [128,135,136]. A comparative overview of the main sequencing technologies, their respective strengths, and limitations is summarized in Table 2.

To maximize the clinical impact of these technologies, ongoing development in bioinformatics and variant interpretation frameworks is essential. Artificial intelligence (AI) is increasingly being integrated to streamline sequencing workflows, optimizing experimental design, predicting outcomes, automating laboratory processes, and supporting large-scale variant analysis [137,138]. This convergence of advanced sequencing and AI-driven analytics represents the next frontier in the precise and timely diagnosis of genetic myopathies.

### 3.2. Emerging Molecular Biomarkers

The development of molecular biomarkers is reshaping how myopathies are monitored, diagnosed, and prognosticated [139]. Traditionally, serum creatine kinase (CK) has been the most widely used marker, but its poor specificity and weak correlation with disease severity limit its usefulness for precision monitoring [140].

Beyond CK, circulating cytokines have gained attention [141]. Recent research has established Growth differentiation factor-15 (GDF-15) as a significant biomarker for mitochondrial myopathies. Its levels are indicative of both disease severity and a patient’s response to metabolic stress. A study has shown that measuring GDF-15 levels can be used to distinguish patients with mitochondrial myopathies from those with other forms of myopathy, suggesting its value in differential diagnosis [142]. Other cytokines, including IL-6 and TNF-α, contribute to the inflammatory profile but lack disease specificity. Because cytokines are central regulators of immune cell activation, differentiation, and recruitment, measuring selected disease-associated cytokines could provide valuable diagnostic information in IIMs, help orient diagnosis toward or away from a genetic myopathy, and assist in preselecting patients in whom a causal gene defect should be sought [141]. In addition, chemokines such as CXCL10 and CCL2 are elevated in IIMs, where they mirror immune cell recruitment and may serve as markers of disease activity and therapeutic response [143,144].

Muscle-specific microRNAs (miRNAs), including miR-1, miR-133a, and miR-206, are released into circulation following muscle degeneration. These molecules have been shown to correlate with disease severity, such as an inverse relationship with NSAA scores in DMD, and may also reflect recovery of muscle integrity after therapeutic interventions [145,146,147,148]. However, a study evaluating these three miRNAs across different muscular dystrophies reported no statistically significant differences between patients and healthy controls after correction for multiple testing, underscoring the need for larger cohorts to validate their diagnostic and prognostic value [149]. Their long-lasting serum stability nonetheless represents an advantage over CK [146].

Beyond miRNAs, muscle-derived proteins such as skeletal troponin I (sTnI), myosin light chain 3 (Myl3), fatty acid-binding protein 3 (FABP3), and creatine kinase muscle-type (CKM) are emerging as dynamic serum biomarkers. These proteins not only display disease-specific profiles but also correlate with clinical endpoints like forced vital capacity in DMD and the 10-m walk test in LGMD R2 [150].

On the other hand, “omics” technologies applied to muscle biopsies, particularly transcriptomics and proteomics, are expanding biomarker discovery by providing detailed molecular signatures of disease [151,152]. Proteomic profiling enables the identification of dysregulated proteins, elucidates the functional impact of genetic variants, and even supports a “protein-first” diagnostic approach by predicting underlying genetic defects. Such analyses capture changes in metabolic pathways, cytoskeletal remodeling, inflammation, and myogenesis, offering a comprehensive view of the pathological state [153,154].

Autoantibodies also represent an important biomarker class in IIMs. Specific myositis-associated autoantibodies are now recognized as identifying distinct, clinically homogeneous subgroups, including five in dermatomyositis, eight in antisynthetase syndrome-associated myositis, two in immune-mediated necrotizing myopathy, and cN1A in inclusion body myositis [155,156,157]. Additionally, novel approaches such as Fourier-transform infrared (FTIR) spectroscopy are being explored as biomarkers in mitochondrial myopathies. FTIR spectroscopy offers a rapid, non-invasive, sensitive, and specific diagnostic tool requiring minimal sample amounts [158].

While these developments are promising, their clinical application varies considerably. Serum CK remains the only routinely used blood biomarker, although its limitations are well recognized [140]. Among circulating proteins, GDF-15 is the most advanced, with multiple studies supporting its diagnostic utility in mitochondrial myopathies [159,160,161], whereas other candidates such as sTnI or FABP3 are still in early validation [150]. Cytokines and chemokines are promising in IIMs, but they currently serve more as research tools than standardized diagnostics [142]. Circulating miRNAs show excellent biological plausibility and stability, yet their use is hampered by a lack of large, harmonized multicenter studies [146,162]. Proteomic and transcriptomic profiles of muscle biopsies are powerful for mechanistic insights and variant interpretation, but their technical complexity, cost, and lack of standardization limit routine use [153]. In contrast, imaging biomarkers, particularly quantitative muscle MRI, are already being incorporated into clinical trials and are beginning to influence patient monitoring in specialized centers [163,164].

Overall, the field is moving beyond non-specific serum enzymes toward disease-tailored biomarkers that capture molecular mechanisms in real time. Circulating proteins, cytokines, chemokines, miRNAs, and omics-based signatures collectively promise to improve patient stratification, enable precise monitoring of therapeutic efficacy, and accelerate the translation of novel therapies into clinical care.

## 4. Clinical Implications of Molecular Advances

The integration of molecular discoveries into clinical practice has fundamentally transformed the classification, prognosis, and management of myopathies [165]. Traditional phenotype-based nosologies are now being replaced by molecularly defined subtypes, providing a more precise and biologically meaningful framework [166].

For example, the LGMDs, once grouped solely by clinical presentation, are now classified by their causative genes (e.g., LGMD R1, LGMD R2), enabling a more accurate understanding of disease mechanisms [32]. Similarly, Duchenne and Becker muscular dystrophies, both caused by mutations in the *DMD* gene, are distinguished by whether the mutation disrupts the reading frame (leading to Duchenne’s severe phenotype) or preserves it (resulting in Becker’s milder course) [13,167]. Myotonic dystrophies are subtyped according to their specific repeat expansions: a (CTG)n repeat in DMPK for DM1 and a (CCTG)n repeat in ZNF9 for DM2 [168]. CMDs have also been redefined at the molecular level, with subtypes linked to mutations in genes such as *LAMA2*, *LARGE*, *FKTN*, *FKRP*, *POMGnT1*, *POMT1*, *POMT2*, and *COL6* [169,170].

These advances are not merely academic; molecular classification directly impacts patient care by guiding therapeutic strategies and improving prognostic accuracy. Mutation type, for instance, is a strong predictor of disease severity: nonsense mutations in *DMD* usually lead to the severe Duchenne phenotype, while in-frame mutations produce the milder Becker form [171,172]. Similarly, molecular findings can predict complications such as cardiac involvement in dystrophinopathies and mitochondrial disorders, enabling earlier surveillance and intervention [173,174,175,176]. Knowledge of a patient’s specific *DMD* mutation, for example, allows cardioprotective therapies to be introduced before the onset of severe cardiomyopathy [175].

Beyond genetics, imaging biomarkers are also refining clinical management. In IIMs, subclinical cardiac involvement can be detected using advanced imaging. Elevated native T1 and T2 mapping values, for instance, correlate with clinical markers of cardiac disease, offering a tool for earlier intervention [177,178]. Likewise, in CMs, histopathological features such as fibrosis and fatty infiltration in muscle biopsies strongly predict disease severity, independent of the underlying mutation [179].

Molecular insights are also transforming clinical trial design through genotype-driven recruitment. This ensures that novel therapies are tested in appropriately stratified populations, maximizing therapeutic efficacy. A prime example is exon-skipping therapy for DMD: eteplirsen specifically targets patients with exon 51 skipping mutations, allowing precision in both trial enrollment and therapeutic application [180,181,182].

## 5. Therapeutic Advances Targeting Molecular Mechanisms

### 5.1. Gene Therapy Strategies

Gene therapy has emerged as one of the most promising approaches for the treatment of myopathies, particularly monogenic disorders such as DMD [10]. Considerable progress has been made in developing gene transfer strategies to restore dystrophin expression, most notably through the use of adeno-associated viral (AAV) vectors, which are safe and non-pathogenic [183].

A major obstacle in DMD gene therapy is the size of the dystrophin gene: its mRNA coding sequence spans ~11.5 kb, exceeding the ~4.5 kb packaging capacity of AAV vectors [184,185]. To overcome this limitation, researchers have engineered abbreviated “mini-” and “micro-dystrophin” constructs that retain essential functional domains of the protein. Insights from BMD, where truncated dystrophins still confer a milder phenotype, support the therapeutic potential of these shortened constructs [186,187]. Multiple recombinant micro-dystrophins with clinical potential have now been generated [186].

Preclinical studies in animal models demonstrate that systemic AAV-mediated micro-dystrophin delivery can ameliorate muscle pathology, improve muscle strength, and reduce cardiomyopathy [188]. However, key challenges remain, including vector delivery to all affected muscles, durability of transgene expression, and immune responses against either the viral capsid or the dystrophin protein itself. High AAV doses can enhance therapeutic benefit but also increase the risk of adverse immune reactions [183,189]. In addition to post-treatment immune responses, pre-existing antibodies against AAV vectors can limit patient eligibility and reduce transduction efficiency. Emerging strategies to overcome this challenge include capsid engineering to evade neutralizing antibodies, transient immunosuppression, and plasmapheresis or antibody-depletion approaches, which are being evaluated in preclinical and early clinical settings [190,191].

In addition to micro-dystrophin transfer, several alternative strategies are under investigation. Stop codon read-through therapies aim to bypass nonsense mutations, which account for ~10–15% of DMD cases, allowing restoration of full-length dystrophin [192,193]. Human artificial chromosome (HAC) technology represents another innovative approach, as HACs can accommodate the entire 79-exon dystrophin gene (~2.4 Mb) without integrating into the host genome. Proof-of-concept studies in chimeric mouse models carrying a dystrophin-HAC demonstrated stable expression of full-length dystrophin in skeletal and cardiac muscle [194,195].

Genome editing approaches, particularly CRISPR/Cas9, offer yet another strategy by enabling targeted correction within the patient’s own genome. Proof-of-concept studies in both cell and animal models of DMD have shown successful restoration of the *DMD* reading frame and dystrophin expression [196]. Advances in viral vector engineering, synthetic gene design, and genome editing have paved the way for mutation-specific interventions, bringing possible therapeutic opportunities for these previously intractable conditions [10]. These strategies are currently at the stage of preclinical validation and early-phase clinical trials, with micro-dystrophin transfer already progressing into multiple Phase I/II studies in DMD [193,197,198].

### 5.2. Antisense Oligonucleotide Therapies

Antisense oligonucleotides (ASOs) are an emerging class of nucleic acid–based drugs that act directly at the RNA level, offering a way to target the root cause of disease rather than downstream effects [199]. These short synthetic molecules bind sequence-specifically to pre-mRNA, where they can modulate splicing to restore the production of functional protein [200].

In DMD, ASOs are designed to induce exon skipping in the *DMD* transcript, thereby restoring the reading frame and enabling production of a truncated but functional dystrophin protein similar to that seen in the milder Becker phenotype [201]. Several ASOs have already reached advanced clinical development. Eteplirsen, golodirsen, and viltolarsen, targeting exons 51 and 53, have received conditional regulatory approval [181,199,201]. Eteplirsen, a phosphorodiamidate morpholino oligomer (PMO), binds to exon 51 and promotes dystrophin restoration. Early clinical studies demonstrated increased dystrophin expression in patients treated with Eteplirsen compared with placebo [202,203]. However, the precise level of dystrophin required to achieve significant long-term functional improvement remains under investigation. Clinical data to date suggest that even modest increases may confer some benefit, but variability between patients and muscle groups indicates that a clear correlation between expression level and functional outcome has yet to be fully established [204,205]. Casimersen, targeting exon 45, has also advanced to late-stage development [206]. Several ASOs, including eteplirsen, golodirsen, viltolarsen, and casimersen, have received conditional regulatory approval in specific DMD subgroups, while next-generation chemistries such as tricyclo-DNA (tcDNA) analogs remain in preclinical and early clinical development [201].

Beyond PMOs, alternative ASO chemistries are being investigated to improve delivery, efficacy, and tissue penetration. For example, tcDNA analogs show promise due to their favorable uptake in multiple tissues and ability to cross the blood–brain barrier, an advantage over some current ASO platforms [206,207].

A major strength of ASO therapy is its mutation-specific precision, allowing for highly targeted treatment. However, this also represents a limitation, as each ASO is effective only for a subset of patients with the corresponding exon mutation. This necessitates the development of a broad pipeline of ASOs to address the diverse mutational spectrum of *DMD*, highlighting the importance of precise molecular diagnostics for patient stratification [208]. Despite these advances, delivery remains a critical challenge. Effective tissue targeting is essential for achieving therapeutic levels of ASOs in skeletal and cardiac muscle, thereby maximizing treatment efficiency [209].

### 5.3. Targeted Pharmacological Interventions

Targeted pharmacological interventions seek to modulate specific pathological pathways uncovered by molecular research [210]. In DMD, the absence of dystrophin initiates a cascade of secondary pathologies, including fibrosis, inflammation, calcium dysregulation, oxidative stress, ischemia, and progressive muscle atrophy [211,212]. While gene- and RNA-based therapies aim to restore dystrophin function, pharmacological strategies focus on mitigating these downstream consequences.

Fibrosis inhibition is a major therapeutic goal, given its central role in disease progression. Agents targeting the TGF-β pathway are under investigation, including losartan, an angiotensin II type 1 receptor blocker that reduces TGF-β expression, and monoclonal antibodies against connective tissue growth factor (CTGF), a key TGF-β effector [213,214].

Anti-inflammatory therapy remains the clinical standard for DMD. Long-term glucocorticoid treatment (using prednisone, prednisolone, deflazacort, or vamorolone) improves muscle strength and delays disease progression primarily through inhibition of the NF-κB pathway [212,215]. Glucocorticoids are also the first-line therapy for IIMs, where they normalize serum muscle enzymes and help preserve muscle strength despite the absence of large controlled trials [212].

Calcium homeostasis modulators are being explored to counteract the pathological Ca^2+^ influx caused by dystrophin deficiency. Calcium channel blockers such as streptomycin and AT-300 have shown variable efficacy across different muscle groups and remain under preclinical and early clinical evaluation [216,217].

Oxidative stress reduction represents another promising avenue, particularly relevant in mitochondrial myopathies but also in dystrophinopathies. Antioxidants under investigation include:

Coenzyme Q10 (CoQ10): shown in clinical trials to enhance mitochondrial respiration and function in patients with mitochondrial cytopathies [218,219].

Idebenone: a synthetic benzoquinone that decreases ROS, improves mitochondrial function, and reduces muscle damage associated with dystrophin deficiency [220].

N-acetylcysteine (NAC): demonstrated in preclinical studies to reduce dystrophic pathology in both skeletal and cardiac muscle of *mdx* mice, suggesting therapeutic potential in DMD [221,222].

Among pharmacological therapies, glucocorticoids remain the only widely approved standard of care, while most anti-fibrotic, calcium-modulating, and antioxidant strategies are still confined to preclinical testing or early-phase clinical trials [212]. Together, these targeted pharmacological approaches complement gene- and RNA-based strategies by addressing the downstream pathological consequences of dystrophin deficiency and other myopathy-related molecular defects.

## 6. Limitations, Ethical Considerations, and Future Directions

Despite remarkable progress in elucidating the molecular underpinnings of myopathies and translating this knowledge into therapeutic strategies, important technical, biological, and ethical challenges continue to shape the trajectory of the field. Current interventions, including gene therapy, antisense oligonucleotides, and targeted pharmacological approaches, have shown encouraging results in slowing or modifying disease progression, yet they rarely achieve complete disease reversal [43,183,184,199,209]. Several factors contribute to this limitation. Immune responses directed against viral vectors or transgene products remain another substantial obstacle, threatening both safety and durability [183]. These technical hurdles mean that therapies that appear promising in controlled experimental settings often demonstrate variable or incomplete efficacy in clinical practice.

The variability in patient responses further complicates clinical application and trial design. Differences in genetic background, including modifier genes and residual protein expression, strongly influence therapeutic outcomes, making it difficult to predict how an individual patient will respond [180,182]. This biological heterogeneity highlights a broader truth: myopathies are not simple “single-gene, single-cure” disorders. Even when the primary genetic defect is targeted, disease progression may continue because of secondary pathological processes such as fibrosis, oxidative stress, chronic inflammation, calcium dysregulation, and impaired regeneration [8]. The interconnected nature of these pathways means that targeting a single mechanism may not suffice, as compensatory cascades can sustain damage. As a result, the current generation of therapies should be considered disease-modifying rather than curative, setting realistic expectations for patients and clinicians alike while underscoring the need for multi-targeted, combinatorial, and personalized approaches.

These technical and biological challenges are mirrored by profound ethical and regulatory concerns. The rapid evolution of gene-based therapies, particularly genome editing technologies such as CRISPR/Cas9, has reignited debates about the acceptable limits of human intervention in genetics [196]. While somatic editing aimed at treating severe neuromuscular disease is widely supported, the potential application of such tools to the germline raises complex questions about heritability, intergenerational impact, and the specter of so-called “designer babies” [223,224]. The promise of repairing fatal gene defects must therefore be weighed carefully against the risks of unintended consequences, both biological and societal.

Even within the realm of accepted clinical practice, equity and access remain pressing concerns. Advanced therapies such as gene transfer or antisense oligonucleotides are extraordinarily expensive to develop and deliver, placing them out of reach for many patients worldwide and creating the risk of widening global health disparities [129,225]. Precision medicine, in its current form, risks becoming accessible only to those in well-resourced healthcare systems, leaving behind patients in low- and middle-income countries [226]. This disparity calls for new funding models, international collaborations, and equitable distribution mechanisms to ensure that groundbreaking therapies do not remain confined to privileged populations.

Compounding these issues is the reality that regulatory frameworks struggle to keep pace with scientific progress. Many approvals for genetic therapies are accelerated on the basis of surrogate molecular endpoints, such as dystrophin expression, rather than long-term clinical outcomes [227]. While this provides patients with earlier access to potentially life-saving therapies, it also raises concerns about safety, durability, and transparency.

At the same time, the field is advancing into new frontiers that hold transformative potential. Artificial intelligence and bioinformatics are emerging as critical tools for integrating vast and complex datasets, ranging from genomics and transcriptomics to imaging and histology [138]. These approaches promise to refine variant interpretation, enhance patient stratification, and predict therapeutic responses with a level of precision previously unattainable. In parallel, dynamic biomarkers are being developed to replace traditional markers such as creatine kinase, which are non-specific and poorly correlated with disease severity [149]. Novel biomarkers, including muscle-specific microRNAs and proteins such as skeletal troponin I, myosin light chain 3, fatty acid-binding protein 3, and muscle-type creatine kinase, are providing more specific, real-time measures of muscle injury and functional status [145,149]. These advances are not only improving monitoring in clinical practice but are also becoming indispensable tools in therapeutic trials, where sensitive, responsive endpoints are critical to evaluating efficacy.

The true promise of these emerging technologies lies in their integration. Artificial intelligence can analyze patterns across dynamic biomarker data, genomic profiles, and imaging results to predict disease progression and optimize therapy in real time [228]. This synergy creates the foundation for an adaptive healthcare system in which patient data continuously refine diagnostic categories, therapeutic choices, and prognostic predictions. Such a paradigm represents a shift from static classifications and trial-and-error management toward dynamic, learning-based precision medicine. In this context, artificial intelligence can support patient selection by integrating genetic variants, molecular biomarkers, clinical trajectories, and imaging features to identify patient subgroups most likely to respond to high-cost or high-risk therapies [229]. Machine learning models can detect response-associated patterns that are not evident through conventional stratification methods, enabling more informed decisions regarding treatment eligibility and timing [230]. Such approaches may reduce unnecessary exposure to ineffective interventions while improving cost-effectiveness and clinical outcomes.

Another important consideration is the treatment and prevention of mitochondrial myopathies. Current management remains largely supportive, focusing on exercise regimens, nutritional supplementation, and metabolic cofactors, although targeted therapies are emerging [231]. Examples include nucleoside supplementation in TK2 deficiency, enzyme replacement in selected mitochondrial depletion syndromes, and early-phase gene or mRNA-based therapies aimed at restoring oxidative phosphorylation [231]. Mitochondrial transplantation and mitophagy modulation are also under investigation as experimental strategies [232]. However, mitochondrial transplantation raises important unresolved questions regarding mitochondrial–nuclear compatibility, potential immunological responses to exogenous mitochondria, and the long-term stability and functional integration of transferred organelles [233]. Beyond therapeutic interventions, preventive approaches have become a reality for mtDNA-encoded defects. Mitochondrial replacement therapy, based on nuclear transfer into donor oocytes with healthy mitochondria, offers the possibility of preventing maternal transmission of pathogenic mtDNA variants [231,234]. While promising, this technology raises profound ethical, regulatory, and societal questions that mirror broader challenges in the field of precision medicine [234].

Overall, while the molecular revolution has profoundly altered the landscape of myopathy research and care, the field now stands at a crossroads where scientific opportunity is tempered by technical limitations, biological complexity, and ethical responsibility. Meeting these challenges will require not only technological innovation but also robust ethical frameworks, equitable access strategies, and interdisciplinary collaboration. The emerging integration of artificial intelligence, bioinformatics, and dynamic biomarkers points toward a future where myopathy management is increasingly personalized and adaptive. Yet the success of this future will depend as much on addressing the societal and ethical dimensions of innovation as on achieving scientific breakthroughs.

## 7. Conclusions

Advances in molecular research have reshaped our understanding of myopathies, moving from descriptive clinical classifications to mechanistic insights that inform precise diagnosis, prognosis, and therapy. The elucidation of genetic and molecular pathways has enabled the development of innovative diagnostic tools, dynamic biomarkers, and mutation-specific treatments such as gene therapy, antisense oligonucleotides, and targeted pharmacological interventions. Yet, despite these remarkable strides, myopathies remain complex disorders driven by multifactorial mechanisms that challenge the notion of a single curative approach.

Recent advances in molecular genetics and immunology have further expanded emerging therapeutic strategies for myopathies. Gene-based approaches, including gene replacement and antisense oligonucleotide therapies, are increasingly complemented by targeted immunomodulatory, metabolic, and mitochondrial interventions, as well as novel pharmacological agents aimed at restoring calcium homeostasis, protein turnover, and muscle regeneration. Although many of these approaches remain disease-modifying rather than curative, they underscore the growing shift toward precision medicine in the management of myopathies.

While the identification of common pathogenic pathways such as calcium dysregulation, mitochondrial dysfunction, or chronic inflammation provides opportunities for broad therapeutic strategies, the diversity of genetic and clinical subtypes requires that such approaches be complemented by mutation-specific or disease-tailored interventions. Current therapies are largely disease-modifying rather than curative, and their success is tempered by technical barriers, biological variability, and ethical and regulatory concerns. Looking ahead, the integration of artificial intelligence, bioinformatics, and novel biomarkers promises to accelerate precision medicine, providing adaptive and personalized strategies for patient care. Realizing this potential will require not only scientific innovation but also equitable access, responsible regulation, and ongoing collaboration across disciplines. Together, these efforts offer a hopeful trajectory toward translating molecular discoveries into meaningful, life-changing outcomes for patients with myopathies.

## Figures and Tables

**Table 1:** Key Genetic Alterations and Pathogenic Mechanisms in Major Myopathy Subtypes.

Myopathy Subtype	Genes Involved	Proteins Affected	Inheritance	Diagnostic Tests/Biomarkers	Key Pathogenic Mechanisms
Dystrophinopathies [13,20,21,24,25,26]	*DMD*	Dystrophin	X-linked. Frameshifting/Nonsense (DMD), In-frame (BMD)	Genetic testing (MLPA, NGS/WGS), muscle MRI/biopsy, CK, cardiac MR	DGC disruption, sarcolemma weakening, functional ischemia, oxidative stress, calcium overloading, regeneration failure
Limb–Girdle Muscular Dystrophies [30,32,34,38,39]	*CAPN3* (LGMD R1)	Calpain 3	Mostly autosomal recessive (R) or dominant (D)	NGS panels/WES/WGS, CK, characteristic muscle MRI patterns	Impaired sarcomeric integrity sensing/repair, disrupted protein turnover
	*DYSF* (LGMD R2)	Dysferlin	Mostly autosomal recessive (R) or dominant (D)	NGS panels/WES/WGS, immunostaining for sarcoglycans/dysferlin, CK, characteristic muscle MRI patterns	Impaired muscle membrane repair
	*SGCA, SGCB *(sarcoglycanopathies; LGMD R3–R4)	α- and β-sarcoglycan	Autosomal recessive	NGS panels/WES/WGS, sarcoglycan immunostaining, elevated CK, characteristic muscle MRI patterns	Destabilization of the dystrophin–glycoprotein complex, sarcolemmal fragility
Congenital Myopathies [44,46,47,52,53,56,57]	*RYR1*	Ryanodine Receptor 1 (RyR1)	Usually autosomal dominant or recessive/de novo	Targeted genetic testing (NGS/WES), muscle biopsy, CK variable	Impaired calcium handling (leaky channels, reduced release), ECC uncoupling
	*SELENON*	Selenoprotein N	Usually recessive/de novo	Targeted genetic testing (NGS/WES), muscle biopsy histology (cores, rods), CK variable	Oxidative stress, altered ECC/calcium homeostasis (secondary *RYR1* impact)
	*ACTA1*	Skeletal muscle α-actin	Usually autosomal dominant	Targeted genetic testing (NGS/WES), muscle biopsy histology (cores, rods), biochemical markers (CK)	Disrupted thin filament function, impaired force generation
Mitochondrial Myopathies [59,61,62,63,68]	mtDNA (e.g., *MT-ND1*, *MT-TL1*) or nDNA	OXPHOS proteins, tRNAs, mtDNA maintenance proteins	Maternal (mtDNA) or autosomal (nuclear)	mtDNA testing, WES/WGS including mtDNA, muscle biopsy (ragged-red fibers, COX-negative), lactate, metabolic testing	Impaired oxidative phosphorylation, ATP deficit, excessive ROS production

Abbreviations: DMD: Duchenne muscular dystrophy; BMD: Becker muscular dystrophy; DGC: Dystrophin glycoprotein complex; ECC: Excitation–contraction coupling; mtDNA: Mitochondrial DNA; nDNA: Nuclear DNA; tRNA: Transfer RNA; OXPHOS: Oxidative phosphorylation; ATP: Adenosine triphosphate; ROS: Reactive oxygen species; CK: Creatine Kinase; NGS: Next-generation sequencing; WES: Whole-exome sequencing.

**Table 2:** Comparison of Sequencing Technologies.

Technology	Scope	Strengths	Limitations	Key References
Sanger Sequencing	Single genes/small regions	High accuracy, gold standard for validation	Low throughput, not suitable for complex/multigene disorders	[123]
Targeted NGS Panels	Known myopathy-related genes	Cost-effective, focused, high sensitivity for selected variants	Misses mutations outside panel	[125,126]
Whole-Exome Sequencing (WES)	Coding regions (~1–2% of genome)	Captures ~85% of disease-causing mutations	Limited detection of intronic/structural variants	[125,126]
Whole-Genome Sequencing (WGS)	Entire genome	Comprehensive, uniform coverage, captures non-coding variants	Expensive, complex data interpretation	[127]
Long-Read Sequencing (LRS)	Long DNA molecules, epigenetic data	Detects STR expansions, CNVs, structural variants, phasing, methylation	Requires high-quality DNA, limited databases, costly	[128,130,132,135,136]

## Data Availability

No new data were created or analyzed in this study. Data sharing is not applicable to this article.
